# Expression of enhancer of zeste homolog 2 correlates with survival outcome in patients with metastatic breast cancer: exploratory study using primary and paired metastatic lesions

**DOI:** 10.1186/s12885-017-3154-3

**Published:** 2017-02-27

**Authors:** Hitoshi Inari, Nobuyasu Suganuma, Kae Kawachi, Tatsuya Yoshida, Takashi Yamanaka, Yoshiyasu Nakamura, Mitsuyo Yoshihara, Hirotaka Nakayama, Ayumi Yamanaka, Katsuhiko Masudo, Takashi Oshima, Tomoyuki Yokose, Yasushi Rino, Satoru Shimizu, Yohei Miyagi, Munetaka Masuda

**Affiliations:** 1 Department of Breast and Endocrine Surgery, Kanagawa Cancer Center, 2-3-2 Nakao, Asahi-ku, Yokohama, 241-0815 Japan; 2 Molecular Pathology and Genetics Division, Kanagawa Cancer Center Research Institute, 2-3-2 Nakao, Asahi-ku, Yokohama, 241-0815 Japan; 3 Department of Pathology, Kanagawa Cancer Center, 2-3-2 Nakao, Asahi-ku, Yokohama, 241-0815 Japan; 4 Department of Surgery, Yokohama City University, 3-9 Fukuura, Kanazawa-ku, Yokohama, 236-0004 Japan

**Keywords:** Metastatic breast cancer, EZH2, Ki-67, Prognostic factor, Immunohistochemistry, Epigenetics

## Abstract

**Background:**

In metastatic breast cancer, the status of the estrogen receptor (ER), progesterone receptor (PR), and human epidermal growth factor receptor 2 (HER2), as well as the Ki-67 index sometimes change between primary and metastatic lesions. However, the change in expression levels of enhancer of zeste homolog 2 (EZH2) between primary and metastatic lesions has not been determined in metastatic breast cancer.

**Methods:**

Ninety-six metastatic breast cancer patients had biopsies or resections of metastatic lesions between September 1990 and February 2014 at the Kanagawa Cancer Center. We evaluated ER, PR, HER2, Ki-67, and EZH2 in primary lesions and their corresponding metastatic lesions using immunohistochemistry. We examined the change in expression of EZH2 between primary and metastatic lesions, the correlation between the expression of EZH2 and the expression of other biomarkers, and the relationship between EZH2 expression and patient outcome in metastatic breast cancer.

**Results:**

EZH2 expression was significantly higher in metastatic lesions compared with primary lesions. EZH2 expression was highly correlated with Ki-67 expression in primary and metastatic lesions. High-level expression of EZH2 was associated with poorer disease-free survival (DFS) outcomes in patients with primary lesions (*P* < 0.001); however, high-level expression of EZH2 was not associated with poorer DFS outcomes in patients with metastatic lesions (*P* = 0.063). High-level expression of EZH2 was associated with poorer overall survival (OS) postoperatively in patients with primary (*P* = 0.001) or metastatic lesions (*P* = 0.005). High-level expression of EZH2 was associated with poorer OS outcomes after recurrence in patients with metastatic lesions (*P* = 0.014); however, high-level expression of EZH2 was not associated with poorer OS outcomes after recurrence in patients with primary lesions (*P* = 0.096). High-level expression of EZH2 in metastatic lesions was independently associated with poorer OS outcomes after recurrence.

**Conclusions:**

EZH2 expression was significantly increased in metastatic lesions compared with primary lesions. High-level expression of EZH2 in metastatic lesions was associated with poorer OS outcomes after primary surgery and recurrence.

**Electronic supplementary material:**

The online version of this article (doi:10.1186/s12885-017-3154-3) contains supplementary material, which is available to authorized users.

## Background

Metastatic breast cancer (MBC) is difficult to treat using currently available conventional therapies, and median long-term survival rates in MBC patients have been reported to be as little as 18–24 months or 2–4 years from the time of diagnosis [1, 2]. Following chemotherapy, 10-year survival rates are approximately 5% and 2–3% in patients with MBC and those who survive >20 years, respectively [3, 4].

Management of MBC generally consists of systemic treatment (chemotherapy and targeted therapy, including anti-estrogen and anti-human epidermal growth factor receptor 2 [HER2] therapies). Treatment decisions for patients with MBC are usually based on estrogen receptor (ER), progesterone receptor (PR) and HER2 status of the primary tumor, the disease-free interval (DFI), site(s) of recurrence and performance status [5]. Because performing biopsies of metastatic lesions risks damaging vital organs and tissues, investigation of biomarkers in metastatic lesions is often challenging. Therefore, based on biomarkers in the primary tumor, the systemic treatment is often given to MBC patients. However, previously published reports show that, because biomarker levels change between primary and metastatic lesions, surgical biopsy of metastatic lesions followed by pathological confirmation for the investigation of biomarkers is occasionally proposed as an effective strategy in the treatment of MBC patients [6–12].

Enhancer of zeste homolog 2 (EZH2) is a well-known histone modifier protein that functions as a methyltransferase at lysine 27 of histone H3 [13]. EZH2 is a member of the polycomb group of genes [14] that is important for transcriptional regulation through chromatin remodeling, nucleosome modification and interactions with other transcription factors. It is assumed that EZH2 promotes breast cancer progression by transcriptional repression of tumor suppressors and by maintaining cells in a stem cell-like state [15, 16]. EZH2 has been demonstrated to be overexpressed in many types of malignancies, including breast, prostate and endometrial cancers, and has been suggested as a candidate for targeted treatment [17, 18]. In primary breast cancer (PBC), Kleer et al. [17] showed that EZH2 overexpression was further associated with a larger tumor size, ER- and PR-negative status, an advanced stage of disease, and significantly reduced disease-free survival (DFS) and overall survival (OS). Other investigators have reported that EZH2 promotes neoplastic progression in the breast, and that downregulation in EZH2 expression reduces in vivo tumor growth of breast cancer cells [17, 19, 20]. EZH2 is important for the control of cell proliferation and invasion, and has recently been shown to regulate DNA repair pathways and genomic stability [19, 21–24]. However, few reports have examined EZH2 expression in metastatic lesions, changes in EZH2 expression levels between primary and metastatic lesions, and patient outcome measures in MBC in relation to EZH2 expression.

The purpose of this study was to examine the expression levels of EZH2 in 96 pairs of primary cancer tissues and metastatic lesions obtained from patients with MBC. To evaluate the clinicopathological significance of EZH2 expression in metastatic lesions, we examined the correlations and changes in ER, PR, HER2, Ki-67 and EZH2 expression between primary cancer tissues and metastatic lesions, and DFS and OS outcomes after primary surgery and recurrence in patients with MBC.

## Methods

### Patients and samples

We retrospectively studied surgical specimens of PBC tumors and their corresponding metastatic lesions from patients who underwent surgery for their PBC tumor at the Kanagawa Cancer Center, Yokohama, Japan, between December 1977 and March 2013. Of those who relapsed after primary surgery between September 1990 and February 2014, there were 96 consecutive patients from whom metastatic lesions were obtained, either by surgery or biopsy, and evaluated using immunohistochemistry (IHC). In all cases, archival hematoxylin and eosin-stained slides of the PBC tumor and its corresponding metastatic lesion were retrieved and reviewed for confirmation of pathological features, as well as to select suitable tissue blocks for IHC analysis. We constructed tissue microarrays (TMAs) using PBC tumors and metastatic lesions. In patients receiving neoadjuvant chemotherapy, we examined the PBC tumor using a core needle biopsy before treatment was commenced in order to avoid potential bias. The Ethics Committees of the Kanagawa Cancer Center, Yokohama, Japan, approved the study protocol.

### TMAs

TMAs consisting of cores, each measuring 2 mm in diameter, were assembled from formalin-fixed, paraffin-embedded blocks of surgically removed tissue from primary tumors and their metastatic lesions in breast cancer patients. We included tissue cores from each primary tumor, metastatic lesion and normal breast tissue, which was used as a control, in the array.

### IHC analysis

IHC staining for biomarkers ER, PR, HER2, Ki-67 and EZH2 was performed in all cases. TMAs were cut into 4-μm-thick sections and mounted onto pre-coated glass slides. All sections were stained using an autostainer (trade name Histostainer; Nichirei Biosciences Inc., Tokyo, Japan) using primary antibodies to ER (clone 1D5, dilution 1:80; Nichirei Biosciences Inc., Tokyo, Japan), PR (clone A9621A, dilution 1:100; Nichirei Biosciences Inc., Tokyo, Japan), HER2 (clone D8F12, dilution 1:800; Cell Signaling Technology Inc., Danvers, MA, USA), Ki-67 (clone SP-6, dilution 1:200; Nichirei Biosciences Inc., Tokyo, Japan) and EZH2 (clone D2C9, dilution 1:50; Cell Signaling Technology Inc., Danvers, MA, USA).

The results of the IHC analysis were assessed in a blinded fashion by a breast surgeon (H.I.) and pathologist (K.K.) who examined each slide independently. Unclear cases were discussed between the breast surgeon and pathologist. Each tumor was assessed twice and an average was calculated between the two scores. Nuclear immunoreactivity of each hormone receptor was scored independently by evaluating the percentage of positively stained cancer cells. ER and PR were defined as positive if there was staining of ≥1% of tumor cell nuclei. HER2 expression was scored as 0, 1+, 2+ or 3+ in accordance with the guidelines of the American Society of Clinical Oncology/College of American Pathologists [25]. A HER2 score of 3+ was considered positive. IHC 2+ tumors were not analyzed using in situ hybridization techniques. A HER2 score of 2+ was considered negative (see Additional file 1).

**Fig. 1:**
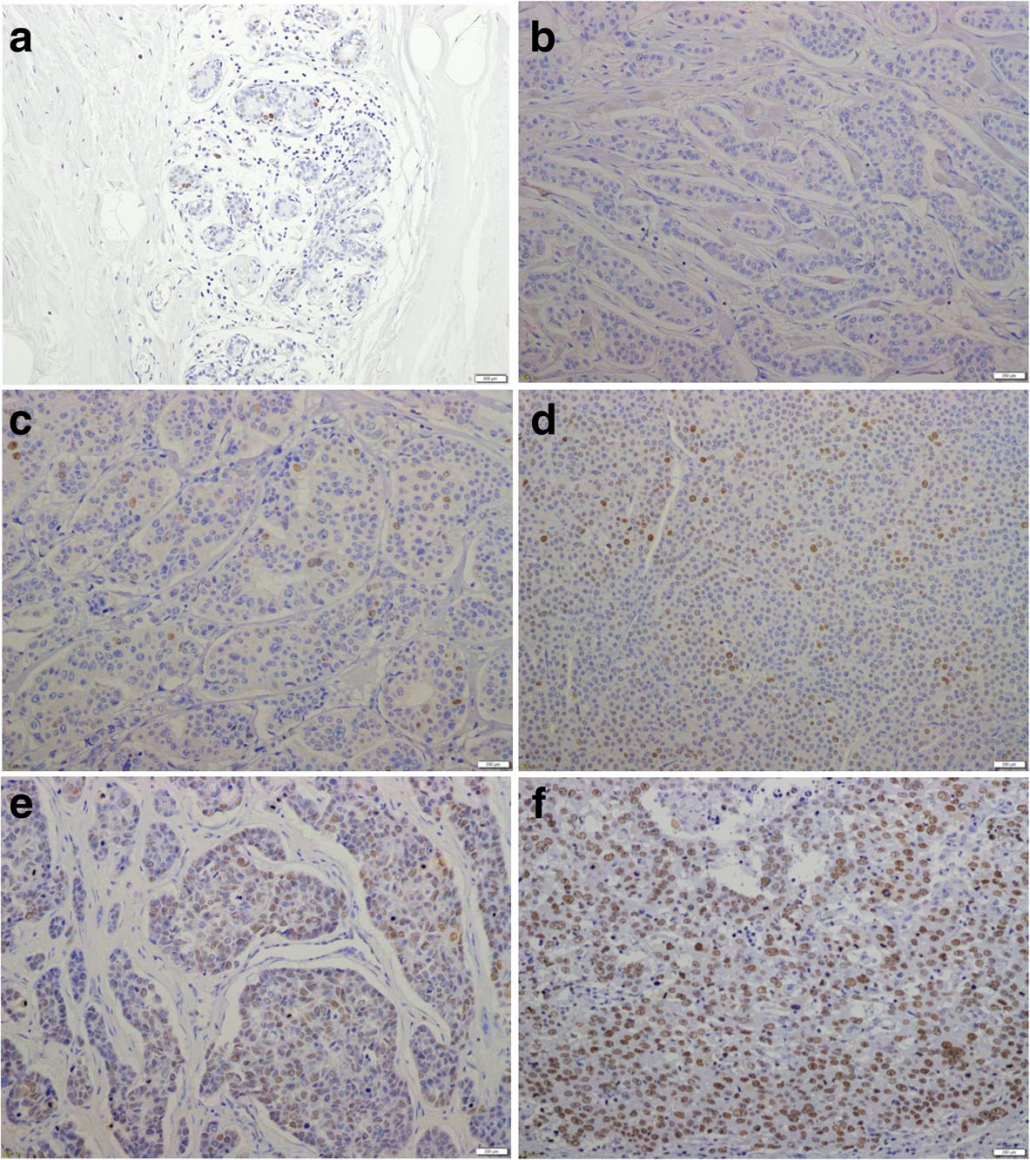
Representative breast tissue sections stained with an antibody to EZH2. Representative examples of primary tissue or metastatic tissue cores presenting with five levels of staining for enhancer of zeste homolog 2 (EZH2):**a** normal breast; **b** ≤1/100 cells stained (Score 1); **c** ≤1/10 cells stained (Score 2); **d** ≤1/3 cells stained (Score 3); **e** ≤2/3 cells stained (Score 4); and **f** >2/3 stained (Score 5) (Original magnification, 200× The under bar is 200 μm.)

Regardless of the staining intensity, nuclear immunoreactivity of EZH2 and Ki-67 expression were scored independently by evaluating the proportion of positively stained cancer cells: Score 1 = ≤1/100 cells stained; Score 2 = ≤1/10 cells stained; Score 3 = ≤1/3 cells stained; Score 4 = ≤2/3 cells stained; and Score 5 > 2/3 cells stained (Fig. 1). EZH2 expression scores of 4 and 5, and Ki-67 expression scores of 3, 4 and 5, were considered high expression. EZH2 expression scores of 1, 2 and 3, and Ki-67 expression scores of 1 and 2 were considered low expression. The median EZH2 score and Ki-67 expression score across all PBC tumors sampled were 4 and 3, respectively (see Additional file 2).

ER, PR, HER2 and Ki-67 expression were used to identify distinct molecular subtypes (Table 1). These were defined as follows: luminal A = ER and/or PR+, HER2−, and low Ki-67 expression; luminal B = ER and/or PR+, HER2−, and high Ki-67 expression; luminal HER2 = ER and/or PR+, HER2+; HER2-type = ER and PR−, HER2+; and triple-negative breast cancer (TNBC) = ER and PR−, HER2 − .

**Table 1:** Comparison of estrogen receptor, progesterone receptor, human epidermal growth factor receptor 2, Ki-67, and enhancer of zeste homolog 2 biomarkers between primary lesions and metastatic lesions in breast cancer patients (*n* = 96)

Biomarker	Primary lesions	Metastatic lesions	*P*-value
ER status, *n* (%)			
positive	51 (53.1)	41 (42.7)	0.149
negative	45 (46.9)	55 (57.3)	
PR status, *n* (%)			
positive	47 (49.0)	39 (40.6)	0.246
negative	49 (51.0)	57 (59.4)	
HER2 status, *n* (%)			
positive	16 (16.7)	14 (14.6)	0.691
negative	80 (83.3)	82 (85.4)	
Ki-67 expression, *n* (%)			
high	55 (57.3)	72 (75.0)	0.010^*^
low	41 (42.7)	24 (25.0)	
EZH2 expression, *n* (%)			
high	54 (56.3)	79 (82.3)	<0.0001^*^
low	42 (43.7)	17 (17.7)	
Molecular subtype, *n* (%)			
luminal A^a^	31 (32.3)	19 (19.8)	0.304
luminal B^b^	22 (22.9)	29 (30.2)	
luminal HER2^c^	5 (5.2)	5 (5.2)	
HER2-type^d^	11 (11.5)	9 (9.4)	
TNBC^e^	27 (28.1)	34 (35.4)	

*Abbreviations*: *ER* estrogen receptor, *EZH2* enhancer of zeste homolog 2, *HER2* human epidermal growth factor receptor 2, *PR* progesterone receptor, *TNBC* triple-negative breast cancer

^a^Luminal A = ER and/or PR+, HER2−, and low Ki-67 expression

^b^Luminal B = ER and/or PR+, HER2−, and high Ki-67 expression

^c^Luminal HER2 = ER and/or PR+, HER2+

^d^HER2-type = ER and PR−, HER2+

^e^TNBC = ER and PR−, HER2−

^*^Indicates values that are statistically significant (*P* < 0.05)

### Follow-up

Follow-up was performed using the KCCH Cancer Registry until October 31, 2015. Active follow-up was conducted by accessing hospital visit records, resident registration cards, and permanent domicile data. During the study period, no subject was lost to follow-up. The day of the biopsy of the metastatic lesions was defined as the date of diagnosis of recurrence. DFS was defined as the period from the day of primary surgery until the day of the biopsy of the metastatic lesions. OS after primary surgery was defined as the period from the day of primary surgery until the day of death. OS after recurrence was defined as the period from the day of biopsy of the metastatic lesions until the day of death. Median follow-up time was 96 months (range, 1–299 months) after the primary operation, and median follow-up time was 40 months (range, 0–231) after recurrence.

### Statistical analyses

Relationships between biomarkers of the primary and metastatic breast cancer lesions and clinicopathological characteristics of the patients were analyzed using chi-square tests. Correlations between EZH2 expression and that of other biomarkers were evaluated using Pearson product-moment correlation coefficients (*r*). EZH2 and Ki-67 scores between primary and metastatic breast cancer lesions were compared using independent *t*-tests. DFS, survival rates after primary surgery, and survival rates after recurrence were analyzed using the Kaplan–Meier method, and any differences in survival rates were assessed using log-rank tests according to the expression of EZH2 in the primary and metastatic lesions. Cox proportional hazards models were applied to the multivariate analyses. Since we showed Ki-67 expression and EZH2 expression in primary and metastatic lesions to be strongly correlated (Table 2), we assessed prognostic factors (except for the Ki-67 expression) in multivariate analysis. For Pearson product-moment correlation coefficients (*r*), a *P* < 0.01, and for chi-square tests, independent *t*-tests, log-rank tests and Cox proportional-hazards models, a *P* < 0.05 was considered statistically significant. All statistical analyses were performed using SPSS version 20 (SPSS Inc., Chicago, IL, USA).

**Table 2:** Correlation coefficient of biomarkers and EZH2 scores

	EZH2	*r*	*P*-value
Primary ER status	Primary	−0.103	0.318
Primary PR status	Primary	−0.111	0.282
Primary HER2 status	Primary	0.361	<0.001^*^
Primary Ki-67 expression	Primary	0.722	<0.0001^*^
Metastatic ER status	Metastatic	−0.099	0.339
Metastatic PR status	Metastatic	−0.190	0.064
Metastatic HER2 status	Metastatic	0.306	0.002^*^
Metastatic Ki-67 expression	Metastatic	0.685	<0.0001^*^

*Abbreviations*: *ER* estrogen receptor, *EZH2* enhancer of zeste homolog 2, *HER2* human epidermal growth factor receptor 2, *PR* progesterone receptor

^*^Indicates values that are statistically significant (*P* < 0.01)

## Results

### Clinicopathological characteristics of all patients with MBC in this study

Patient characteristics are summarized in Table 3. Among 96 biopsies or resections of metastases, 26 (27.0%) were of brain, eight (8.3%) of lung, one (1.0%) of liver, two (2.0%) of ovary, 13 (13.5%) of chest wall, 15 (15.6%) of lymph nodes, seven (7.3) of distant skin and 24 (25%) of bone.

**Table 3:** Clinicopathological Characteristics of all metastatic patients in this study

Characteristic	Patients (*n* = 96)	Percent
Age, years (mean ± SD)	51 ± 7.5	
Menopausal status		
pre-	44	45.8
post-	52	54.2
Tumor size		
≤ 20 mm	17	17.7
> 20 mm	77	80.2
unknown	2	2.1
LN status		
positive	63	65.6
negative	33	34.4
Histological type		
ductal	83	86.5
special^a^	13	13.5
LVI status		
positive	63	65.6
negative	25	26.1
unknown	8	8.3
Operation status		
partial mastectomy	17	17.8
mastectomy	79	82.2
Stage at the primary diagnosis		
1	11	11.5
2	48	50
3	34	35.4
4	1	1.0
unknown	2	2.0
Chemotherapy		
adjuvant	83	86.5
none	13	13.5
Hormone therapy		
adjuvant	50	52.1
none	46	47.9
Site of recurrence		
brain	26	27.0
lung	8	8.3
liver	1	1.0
ovary	2	2.1
chest wall	13	13.5
lymph node	15	15.6
distant skin	7	7.3
bone	24	25

*Abbreviations*: *LN* lymph node, *LVI* lymphovascular invasion, *SD* standard deviation

^a^Special type is invasive breast carcinoma except invasive ductal carcinoma

### Changes in ER, PR, HER2, Ki-67 and EZH2 expression between primary and metastatic lesions

**Fig. 2:**
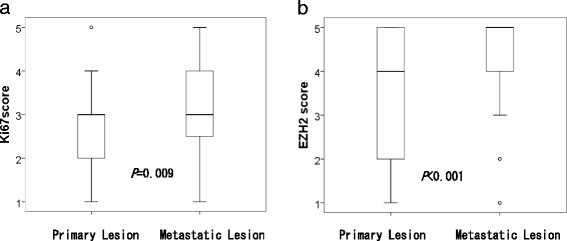
Comparison of the Ki67 expression score and EZH2 expression between primary and metastatic lesions. The mean and standard deviation score of (**a**) Ki67 expression in primary lesions was 2.74 ± 0.92, and in metastatic lesions was 3.10 ± 0.97. The mean and standard deviation score of (**b**) EZH2 expression in primary lesions was 3.56 ± 1.34, and in metastatic lesions was 4.26 ± 1.08

Compared with primary lesions, metastatic lesions exhibited significantly higher levels of expression of Ki-67 (75.0% vs. 57.3% *P* = 0.010) and EZH2 (82.3% vs. 56.3% *P* < 0.0001). Conversely, no statistical differences in ER (42.7% vs. 53.1%, *P* = 0.149), PR (40.6% vs. 49.0%, *P* = 0.246) or HER2 status (14.6% vs. 16.7%, *P* = 0.691) were observed between primary and metastatic lesions (Table 1). We subsequently analyzed the scores of expression levels of Ki-67 and EZH2, which demonstrated a significant difference between the two groups (i.e., high vs. low expression) (Fig. 2). The means and standard deviations of the Ki-67 scores were 2.74 ± 0.92 and 3.10 ± 0.97 for primary and metastatic lesions, respectively (*P* = 0.009) (Fig. 2a), while the means and standard deviations of the EZH2 expression scores were 3.56 ± 1.34 and 4.26 ± 1.08 for primary and metastatic lesions, respectively (*P* < 0.001) (Fig. 2b). Ki-67 and EZH2 expression scores were significantly higher in metastatic lesions compared with PBC lesions.

### Correlation coefficients of ER, PR, HER2, Ki-67 and EZH2 expression scores in primary and metastatic lesions

In PBC lesions, ER (*r* = −0.103, *P* = 0.318) and PR (*r* = −0.111, *P* = 0.282) status were not significantly correlated with EZH2 expression, HER2 status exhibited a significant low correlation with EZH2 expression (*r* = 0.361, *P* < 0.001), and Ki-67 expression exhibited a significant high correlation with EZH2 expression (*r* = 0.722, *P* < 0.0001) (Table 2). Similarly, in metastatic lesions, ER (*r* = −0.099, *P* = 0.339) and PR (*r* = −0.190, *P* = 0.064) status were not significantly correlated with EZH2 expression, HER2 status exhibited a significant low correlation with EZH2 expression (*r* = 0.306, *P* = 0.002), and Ki-67 expression scores exhibited a high correlation with EZH2 expression (*r* = 0.685, *P* < 0.001) (Table 2).

### Relationship between EZH2 expression and patient clinicopathological characteristics

Patient clinicopathological characteristics and their correlation with EZH2 expression in primary and metastatic lesions are summarized in Table 4. Relationships between the expression level status of EZH2 and patient age, menopausal status, tumor size, lymph node status, histological type (i.e., ductal vs. special), lymphovascular invasion status, operation status (i.e., partial vs. full mastectomy), adjuvant chemotherapy and hormone therapy status, primary ER status, primary PR status, primary HER2 status, primary Ki-67 expression, metastatic ER status, metastatic PR status, metastatic HER2 status, metastatic Ki-67 expression, the site(s) of recurrence (i.e., viscera, soft tissue or bone) and DFI (disease-free interval) (i.e., ≤2 years, >2 years, ≤10 years, >10 years) were evaluated. Factors significantly associated with PBC lesions included lymph node status, histological type, primary ER status, metastatic PR status, primary and metastatic HER2 status, primary and metastatic Ki-67 expression, and DFI, whereas factors significantly associated with metastatic lesions included histological type, adjuvant chemotherapy status, primary PR status, primary HER2 status, primary and metastatic Ki-67 expression, and the site(s) of recurrence (Table 4).

**Table 4:** Relationship betweenEZH2 expression in primary and metastatic breast cancer lesions and the clinicopathological characteristics of patients (*n* = 96)

Characteristic	Primary EZH2		*P-*value	Metastatic EZH2		*P*-value
	low (*n* = 42)	high (*n* = 54)		low (*n* = 17)	high (*n* = 79)	
Age, years (mean ± SD)	52 ± 8.5	50 ± 9.8	0.198	50 ± 7.8	51 ± 9.7	0.241
Menopausal status, *n* (%)						
pre-	17 (38.6)	27 (61.4)	0.353	10 (22.7)	34 (77.3)	0.236
post-	25 (48.1)	27 (51.9)		7 (13.5)	45 (86.5)	
Tumor size, *n* (%)						
≤ 20 mm	8 (47.1)	9 (52.9)		2 (11.8)	15 (88.2)	
> 20 mm	33 (42.9)	44 (57.1)	0.936	14 (18.2)	63 (81.8)	0.396
unknown	1 (50.0)	1 (50.0)		1 (50.0)	1 (50.0)	
LN status, *n* (%)						
positive	23 (36.5)	40 (63.5)		11 (17.5)	52 (82.5)	
negative	19 (57.6)	14 (42.4)	0.048^*^	6 (18.2)	27 (81.8)	0.930
Histological type*, n* (%)						
ductal	32 (38.6)	51 (61.4)	0.010^*^	12 (14.5)	71 (85.5)	0.035^*^
special	10 (76.9)	3 (23.1)		5 (38.5)	8 (61.5)	
LVI status, *n* (%)						
positive	32 (50.8)	31 (49.2)		11 (17.5)	52 (82.5)	
negative	8 (32.0)	17 (68.0)	0.148	4 (16.0)	21 (84.0)	0.842
unknown	2 (25.0)	6 (75.0)		2 (25.0)	6 (75.0)	
Operation status, *n* (%)						
partial mastectomy	4 (23.5)	13 (76.5)	0.064	2 (11.8)	15 (88.2)	0.479
mastectomy	38 (48.1)	41 (51.9)		15 (19.0)	64 (81.0)	
Chemotherapy, *n* (%)						
adjuvant	34 (41.0)	49 (59.0)	0.164	12 (14.5)	71 (85.5)	0.035^*^
none	8 (61.5)	5 (38.5)		5 (38.5)	8 (61.5)	
Hormone therapy, *n* (%)						
adjuvant	26 (52.0)	24 (48.0)	0.089	12 (24.0)	38 (76.0)	0.092
none	16 (34.8)	30 (65.2)		5 (10.9)	41 (89.1)	
Primary ER status, *n* (%)						
positive	28	23	0.019^*^	12	39	0.112
negative	14	31		5	40	
Primary PR status, *n* (%)						
positive	24	23	0.157	12	35	0.049^*^
negative	18	31		5	44	
Primary HER2 status, *n* (%)						
positive	1	15	0.001^*^	0	16	0.042^*^
negative	41	39		17	63	
Primary Ki-67 expression, *n* (%)						
high	8	47	<0.001^*^	2	53	<0.001^*^
low	34	7		15	26	
Metastatic ER status, *n* (%)						
positive	21	20	0.203	8	33	0.689
negative	21	34		9	46	
Metastatic PR status, *n* (%)						
positive	22	17	0.039^*^	9	30	0.254
negative	20	37		8	49	
Metastatic HER2 status, *n* (%)						
positive	1	13	0.003^*^	0	14	0.060
negative	41	41		17	65	
Metastatic Ki-67 expression, *n* (%)						
high	23	49	<0.001^*^	2	70	<0.001^*^
low	19	5		15	9	
Site of recurrence, *n* (%)						
viscera^b^	14 (37.8)	23 (62.2)	0.102	5 (13.5)	32 (86.5)	0.012^*^
soft tissue^c^	13 (37.1)	22 (62.9)		3 (8.6)	32 (91.4)	
bone	15 (62.5)	9 (37.5)		9 (37.5)	15 (62.5)	
Disease free interval						
≤ 2 years	7 (23.5)	24(77.4)	0.04^*^	4 (12.9)	27 (87.1)	0.394
> 2 years	35 (53.8)	30 (46.1)		13 (20)	52 (80)	
≤ 10 years	32 (38.6)	51 (61.4)	0.010^*^	13 (15.7)	70 (84.3)	0.185
> 10 years	10 (76.9)	3 (23.1)		4 (30.8)	9 (69.2)	

*Abbreviations*: *ER* estrogen receptor, *EZH2* enhancer of zeste homolog 2, *HER2* human epidermal growth factor receptor 2, *LN* lymph node, *LVI* lymphovascular invasion, *PR* progesterone receptor, *SD* standard deviation

^a^Special type is invasive breast carcinoma except invasive ductal carcinoma

^b^Viscera includes brain, lung, liver and ovary

^c^Soft tissues includes chest wall, lymph node, distant skin

^*^Indicates values that are statistically significant (*P* < 0.05)

### Relationship between EZH2 expression and patient outcome

**Fig. 3:**
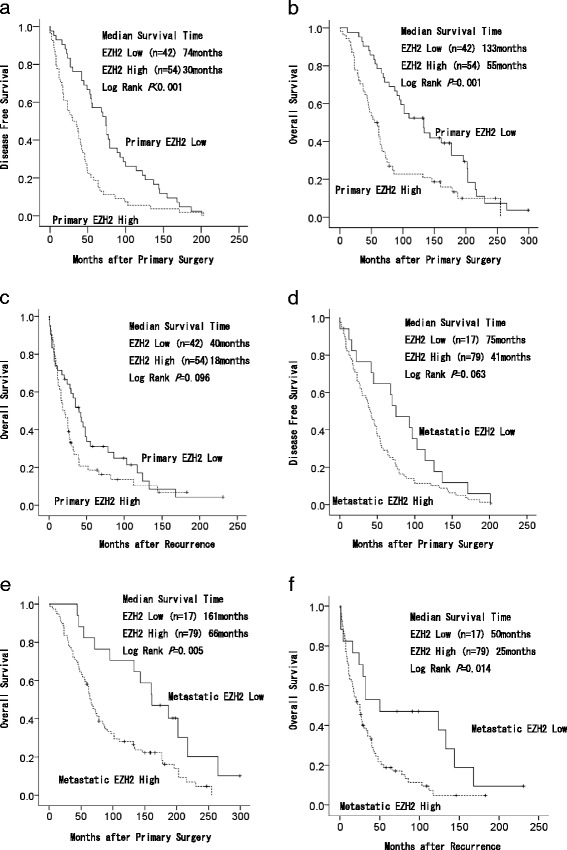
Kaplan–Meier survival curves for breast cancer patients (*n* = 96) with high and low enhancer of zeste homolog 2 (EZH2) expression. Kaplan–Meier survival curves for breast cancer patients with high and low enhancer of zeste homolog 2 (EZH2) expression in (**a**) disease-free survival in patients with primary lesions, **b** overall survival in patients after primary surgery with primary lesions, **c** overall survival in patients after recurrence with primary lesions, **d** disease-free survival in patients with metastatic lesions, **e** overall survival in patients after primary surgery with metastatic lesions, and (**f**) overall survival in patients after recurrence with metastatic lesions

We examined DFS and OS outcomes after primary surgery and recurrence in patients with MBC according to primary EZH2 expression (Fig. 3a, b, c), and DFS and OS outcomes after primary surgery and recurrence in patients with MBC according to metastatic EZH2 expression (Fig. 3d, c, f). Patient clinicopathological characteristics and their correlation with EZH2 expression in primary and metastatic lesions are summarized in Table 4. First, DFS, survival rates after primary surgery, and survival rates after recurrence were analyzed according to the expression of EZH2 in PBC lesions. Low EZH2 expression in PBC lesions occurred in 42 patients and high EZH2 expression in PBC lesions in 54 patients. Median DFS time in patients with high expression levels of EZH2 in PBC lesions was 30 compared with 74 months in patients with low expression levels of EZH2 in PBC lesions (Fig. 3a). Median survival time after primary surgery in patients with high expression levels of EZH2 in PBC lesions was 55 compared with 133 months in patients with low expression levels of EZH2 in PBC lesions (Fig. 3b). Patients expressing high levels of EZH2 in PBC lesions had significantly poorer DFS and OS outcomes than patients expressing low levels of EZH2 in PBC lesions (*P* < 0.001, *P* = 0.001). Second, DFS, survival rates after primary surgery, and survival rates after recurrence were analyzed according to the expression of EZH2 in the metastatic lesions. Low EZH2 expression in metastatic lesions occurred in 17 patients and high EZH2 expression in metastatic lesions occurred in 79 patients. Median survival time after primary surgery in patients with high expression levels of EZH2 in metastatic lesions was 66 compared with 161 months in patients with low expression levels of EZH2 in metastatic lesions (Fig. 3e). Median survival time after recurrence in patients with high expression levels of EZH2 in metastatic lesions was 25 compared with 50 months in patients with low expression levels of EZH2 in metastatic lesions (Fig. 3f). Patients expressing high levels of EZH2 in metastatic lesions had significantly poorer OS outcomes after primary surgery and recurrence than patients expressing low levels of EZH2 in metastatic lesions (*P* = 0.005, *P* = 0.014).

In the univariate analysis, compared with low EZH2 expression, high EZH2 expression was not a poor prognostic indicator of OS after recurrence outcome in PBC (hazard ratio [HR] 1.449; 95% confidence interval [CI] 0.930–2.258, *P =* 0.101) (Table 5), whereas in metastatic lesions, high EZH2 expression was a poor prognostic indicator of OS outcome after recurrence (HR 2.116; 95% CI 1.143–3.916, *P* = 0.017) (Table 5). Other poor prognostic indicators of OS outcome after recurrence in PBC and metastatic lesions from the univariate analysis included primary ER or primary PR status, primary and metastatic high Ki-67 expression, and ≤2 years of DFI (Table 5). A Cox proportional-hazards model using multivariate analysis but not including Ki-67 expression demonstrated that high EZH2 expression was independently associated with poorer OS outcomes after recurrence in patients with metastatic lesions (HR 2.047; 95% CI 1.074–3.902, *P* = 0.029) (Table 5). Multivariate analysis of prognostic factors related to OS after recurrence including Ki-67 expression is shown in Additional file 3: Table S1.

**Table 5:** Univariate and multivariate analysis of prognostic factors related to overall survival after recurrence (*n* = 96)

Prognostic factor	Patients (*n* = 96)	Univariate analysis			Multivariate analysis		
		HR	95% CI	*P*-value	HR	95% CI	*P*-value
Menopausal status, *n* (%)							
pre-	44 (45.8)	1					
post-	52 (54.2)	1.396	0.891–2.186	0.145			
Tumor size, *n* (%)							
≤ 20 mm	17 (17.7)	1					
> 20 mm	77 (80.2)	1.317	0.726–2.388	0.364			
unknown	2 (2.1)						
LN status, *n* (%)							
positive	63 (65.6)	1.309	0.829–2.066	0.248			
negative	33 (34.4)	1					
Histological type, *n* (%)							
ductal	83 (86.5)	1					
special^a^	13 (13.5)	1.028	0.543–1.945	0.932			
LVI status, *n* (%)							
positive	63 (65.6)	1.287	0.769–2.156	0.337			
negative	25 (26.1)	1					
unknown	8 (8.3)						
Operation status, *n* (%)							
partial mastectomy	17 (17.7)	1					
mastectomy	79 (82.3)	0.894	0.500–1.596	0.704			
Chemotherapy, *n* (%)							
adjuvant	83 (86.5)	1.320	0.707–2.461	0.383			
none	13 (13.5)	1					
Hormone therapy, *n* (%)							
adjuvant	50 (52.1)	0.719	0.460–1.122	0.146			
none	46 (47.9)	1					
Primary ER status, *n* (%)							
positive	51 (53.1)	0.541	0.344–0.850	0.008^*^	0.724	0.382–1.372	0.322
negative	45 (46.9)	1					
Primary PR status, *n* (%)							
positive	47 (49.0)	0.595	0.380–0.931	0.023^*^	0.911	0.484–1.714	0.773
negative	49 (51.0)	1					
Primary HER2 status, *n* (%)							
positive	16 (16.7)	1.122	0.616–2.046	0.706			
negative	80 (83.3)	1					
Primary Ki-67 expression, *n* (%)^b^							
high	55 (57.3)	1.848	1.170–2.917	0.008^*^			
low	41 (42.7)	1					
Primary EZH2 expression, *n* (%)							
high	54 (56.3)	1.449	0.930–2.258	0.101			
low	42 (43.7)	1					
Metastatic ER status, *n* (%)							
positive	41 (42.7)	0.972	0.625–1.513	0.901			
negative	55 (57.3)	1					
Metastatic PR status, *n* (%)							
positive	39 (40.6)	0.692	0.440–1.089	0.112			
negative	57 (59.4)	1					
Metastatic HER2 status, *n* (%)							
positive	14 (14.6)	1.282	0.673–2.440	0.450			
negative	82 (85.4)	1					
Metastatic Ki-67 expression, *n* (%)^b^							
high	72 (75.0)	2.422	1.394–4.206	0.002^*^			
low	24 (25.0)	1					
Metastatic EZH2 expression, *n* (%)							
high	59 (77.6)	2.116	1.143–3.916	0.017^*^	2.047	1.074–3.902	0.029^*^
low	17 (22.4)	1					
Metastatic sites, n (%)							
bones	24 (25.0)	0.899	0.548–1.476	0.674			
others	72 (75.0)	1					
Disease free interval							
≤ 2 years	28 (29.0)	1.713	1.086–2.702	0.021^*^	1.601	0.978–2.621	0.061
> 2 years	68 (71.0)	1					
≤ 10 years	83 (87.0)	1					
> 10 years	13 (13.0)	0.861	0.442–1.679	0.661			

*Abbreviations*: *CI* confidence interval, *ER* estrogen receptor, *EZH2* enhancer of zeste homolog 2, *HER2* human epidermal growth factor receptor 2, *HR* hazard ratio, *LN* lymph node, *LVI* lymphovascular invasion, *PR* progesterone receptor

^a^Special type is invasive breast carcinoma except invasive ductal carcinoma

^b^Ki-67 expression was excluded from the multivariate analysis because of an association with EZH2 expression in primary and metastatic breast cancer lesions

^*^Indicates values that are statistically significant (*P* < 0.05)

## Discussion

In this study, using primary and paired metastatic lesions from patients with MBC, EZH2 expression scores correlated significantly with Ki-67 expression scores in both primary and metastatic lesions, and Ki-67 expression and EZH2 expression scores were significantly higher in metastatic lesions compared with PBC lesions. Because Ki-67 expression scores in metastatic lesions increased more than in PBC lesions, we considered that proliferation in metastatic lesions increased more than in PBC lesions. We expected that EZH2 expression in metastatic lesions would be higher than that in PBC lesions. We showed that EZH2 expression correlated significantly with the Ki-67 expression in both PBC and metastatic lesions. We thought that EZH2 promotes breast cancer progression by transcriptional repression of tumor suppressors; consequently, Ki-67 expression increased in breast cancer cells with high EZH2 expression. We showed that EZH2 expression levels in bone metastatic lesions were significantly lower than in viscera and soft tissue metastatic lesions. Expression of EZH2 in primary tumors of patients with DFIs ≤2 years was higher than in those with DFIs >2 years, whereas expression of EZH2 in primary tumors of patients with DFIs >10 years was lower than in those with DFIs ≤10 years. Given that bone metastasis occurs more frequently in ER-positive than ER-negative breast cancer [26] and in MBC patients with DFIs >10 years [27], we expected that EZH2 expression would be low in bone metastasis and in MBC patients with DFIs >10 years. However, because MBC patients with DFIs ≤2 years had a poor prognosis, we expected that EZH2 expression levels would be higher. We showed that high EZH2 expression in primary lesions was shown to be independently associated with poorer DFS and OS outcomes after primary surgery in MBC, whether or not high EZH2 expression in primary lesions was shown to be associated with OS after recurrence. High EZH2 expression in metastatic lesions was not associated with DFS after primary surgery, even if high EZH2 expression in metastatic lesions was shown to be independently associated with poorer OS outcomes after primary surgery and recurrence. We had considered the proliferation of primary lesions associated with DFS and OS after primary surgery until recurrence; on the other hand, proliferation of metastatic lesions was more associated with OS after recurrence than PBC lesions.

Previous reports have demonstrated that in PBC, EZH2 expression was significantly increased in malignant tumors, and was associated with a larger tumor size, ER- and PR-negative status, TNBC, advanced stage disease and reduced progression-free survival and OS [17, 28, 29]. In colon cancer and poorly differentiated synovial sarcomas, EZH2 expression was significantly related to increased tumor cell proliferation, as assessed using the Ki-67 expression [30, 31]. Nishimura et al. [12] reported that, in comparison to primary lesions, the Ki-67 expression score increased significantly in metastatic lesions. We found that in breast cancer patients, EZH2 expression scores correlated significantly with Ki-67 expression scores in both primary and metastatic lesions and Ki-67 expression and EZH2 expression scores were significantly higher in metastatic lesions compared with PBC lesions. Furthermore, high EZH2 expression in metastatic lesions was shown to be independently associated with poorer OS outcomes after recurrence in MBC. Few reports have examined the relationship between EZH2 expression in metastatic lesions and outcomes in patients with MBC.

MBC is difficult to treat using conventional therapies that are currently available on the market, and development of new therapeutic approaches is needed. Considering the downstream effects of EZH2, silencing of the EZH2 gene in the ER+ MCF-7 cell line resulted in higher expression of ER and increased sensitivity to anti-estrogen therapy [32]. EZH2 gene silencing has also been reported to result in a significant reduction in tumor growth in the MB-231 TNBC orthotopic mouse model of breast carcinomas. High EZH2 expression was shown to be significantly associated with TNBC and reduced OS outcomes [28]. In our cohort of MBC patients, we demonstrated a significant correlation between EZH2 expression and Ki-67 expression scores in primary and metastatic lesions. Therefore, EZH2 may represent a potential therapeutic target for this aggressive breast cancer that exhibits high expression levels of Ki-67, thus warranting further investigation. Using data obtained in this study as a reference of expression of EZH2 status in metastatic lesions and the correlation between EZH2 and other biomarkers in MBC, biopsy of metastatic lesions may become less necessary, thereby avoiding risk of vital organ damage because of the biopsy procedure.

Recently, several EZH2 inhibitors have been developed and tested in multiple types of cell lines and xenografts [33, 34]. Both EPZ-6438 (E7438) and GSK126, selective small-molecule inhibitors of histone methyltransferase activity, have yielded promising results in small cell lung cancer cell lines and malignant rhabdoid tumors [33, 34]. In the second quarter of 2015, Epizyme® Inc., Cambridge, MA, USA initiated a phase 2 monotherapy trial of EPZ-6438 in patients with relapsed or refractory non-Hodgkin lymphomas [35].

This study has some limitations. First, the retrospective nature of the study design was prone to selection bias. Patients in this study tended to have a more aggressive breast cancer with poorer prognosis in comparison with a group from the general breast cancer patient population, since all of those recruited were diagnosed with MBC. Second, this study could not consider the effects of adjuvant therapy, which differed according to each patient, owing to the fact that patients were recruited over a long period, from 1977 to 2013. Previous reports have demonstrated that examining change due to treatment based on Ki-67 expression, the number of responders to endocrine therapy as the neoadjuvant therapy declined, and the prognosis of patients exhibiting decreased levels of Ki-67 was good [36]. In addition, the prognosis of patients with decreased levels after chemotherapy was also reported to be good [37, 38]. Third, the EZH2 scoring method in this study was used in house as described in the Materials and Methods section. Forth, we could not assess EZH2 expression in this study according to subtype (luminal A, luminal B, luminal HER2, HER2-type and triple-negative breast cancer) due to sample limitation. We assessed the clinicopathological significance of EZH2 expression in MBC according to subtype; however, the number of breast cancer patients for each subtype was too small to show statistical significance. Increasing the number of MBC patients in this study was not possible since we had already collected samples from 96 patients between 1977 and 2013, which was the maximum that was achievable. Fifth, we used a HER2 immunostaining score of 2+ to designate a negative score, as described in the Materials and Methods section and Additional file 1.

As further investigations, we think that examination of the correlation between EZH2 expression levels and pathological response, and the correlation between EZH2 expression levels and prognosis in neoadjuvant chemotherapy and hormonal therapy should be considered.

## Conclusion

Our results suggest that EZH2 expression levels correlate significantly with the Ki-67 expression score. Therefore, EZH2 may represent a potential therapeutic target for this aggressive breast cancer, especially for those with a high Ki-67 expression score, which warrants further investigation. EZH2 expression scores were significantly higher in metastatic lesions compared with PBC lesions. We also showed that high EZH2 expression levels in primary lesions were independently associated with poorer DFS and OS after primary surgery, and that high EZH2 expression levels in metastatic lesions were independently associated with poorer OS outcomes after primary surgery and recurrence. A part of the present work was precedingly reported at the 2016 ASCO Annual Meeting [39].

## Additional files

Additional file 1: Reasons for assessing cases scored as HER2 2+ as negative. (DOC 23 kb)
Additional file 2: Evaluation of immunostaining of EZH2 and Ki67. (DOC 52 kb)
Additional file 3: Table S1. Univariate and multivariate analysis of prognostic factors related to overall survival after recurrence including Ki-67 expression. (DOC 111 kb)
Additional file 4: Dataset of this study. (XLSX 26 kb)

## Abbreviations

CI: Confidence interval
DFI: Disease-free interval
DFS: Disease-free survival
ER: Estrogen receptor
EZH2: Enhancer of zeste homolog 2
HER2: Human epidermal growth factor receptor 2
HR: Hazard ratio
IHC: Immunohistochemistry
MBC: Metastatic breast cancer
OS: Overall survival
PBC: Primary breast cancer
PR: Progesterone receptor
TMA: Tissue microarray
TNBC: Triple-negative breast cancer
