# Supplementary material for: Expression of enhancer of zeste homolog 2 correlates with survival outcome in patients with metastatic breast cancer: exploratory study using primary and paired metastatic lesions
Source: BMC Cancer. 2017 Feb 27;17:160. doi: 10.1186/s12885-017-3154-3 (PMC5330119; doi:10.1186/s12885-017-3154-3)
Supplement: Additional file 1: — Reasons for assessing cases scored as HER2 2+ as negative. (DOC 23 kb) [file 12885_2017_3154_MOESM1_ESM.doc]

**Additional file 1.**

**Reasons for assessing cases scored as HER2 2+ as negative**

A previous report compared HER2 scoring using IHC data generated with CB11 and the Hercep Test versus IHC data and FISH from the TMAs [1]. Of the 46 HER2 score of 2+ cases on TMA slides using CB11, there were only 8 (17%) cases that showed HER2 gene amplification using FISH, and of the 34 HER2 score of 2+ cases on TMA slides using Hercep Test, there were 12 (35%) cases that showed HER2 gene amplification using FISH. Therefore, we used a HER2 immunostaining score of 2+ to designate a negative score in this study and did not use FISH to evaluate gene amplification.

1. Egervari K1, Szollosi Z, Nemes Z. Tissue microarray technology in breast cancer HER2 diagnostics. Pathol Res Pract. 2007;203(3):169-77. Epub 2007 Feb 9.
