# Supplementary material for: Expression of enhancer of zeste homolog 2 correlates with survival outcome in patients with metastatic breast cancer: exploratory study using primary and paired metastatic lesions
Source: BMC Cancer. 2017 Feb 27;17:160. doi: 10.1186/s12885-017-3154-3 (PMC5330119; doi:10.1186/s12885-017-3154-3)
Supplement: Additional file 2: — Evaluation of immunostaining of EZH2 and Ki67. (DOC 52 kb) [file 12885_2017_3154_MOESM2_ESM.doc]

**Additional file 2.**

**Evaluation of immunostaining of EZH2 and Ki67**

Several studies reported classification in immunostaining of EZH2, however there is no standard classification in immunostaining of EZH2 in TMAs [1,2]. For us to objectively determine, we used the proportion score of Allred Score (Score 1 = ≤1/100 cells stained; Score 2 = ≤1/10 cells stained; Score 3 = ≤1/3 cells stained; Score 4 = ≤2/3 cells stained; and Score 5 >2/3 cells stained) to evaluate the immunostaining of EZH2 [3]. The Allred Score is commonly used in immunohistochemical analysis of breast cancer. We show that immunostaining score of primary EZH2 in the histogram. The histogram show the number of patients on the vertical axis, and immunostaining score on the horizontal axis. The median EZH2 score across all PBC tumors sampled was 4. EZH2 expression scores of 4 and 5 were considered high expression. EZH2 expression scores of 1, 2 and 3 were considered low expression. Several studies showed that the rate of high EZH2 expression in PBC lesions was almost half [1], and we showed that the rate of high EZH2 expression in PBC lesions was 56% in this study. We considered, therefore, that the evaluation of immunostaining of EZH2 in this study was consistent with the previous study.


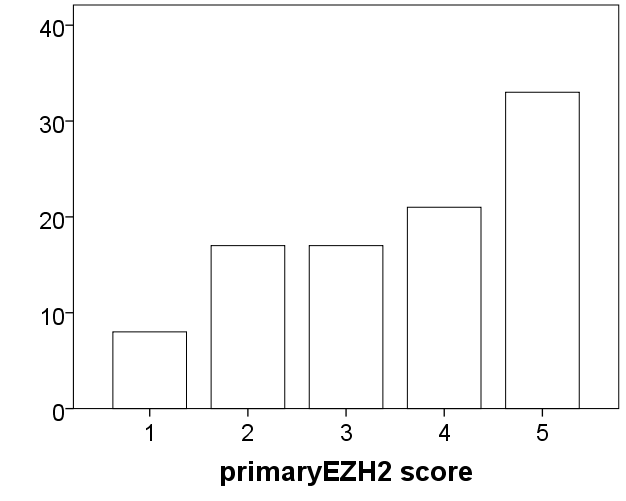


Patient number

The International Ki67 in Breast Cancer Working Group recommended that Ki67 labeling expression should be visually scored for the percentage of tumor cell nuclei with positive immunostaining above the background level, and that at least 500 invasive carcinoma cells should be counted for each case [4]. However, although we assessed TMAs in all cases, we were unable to carry out this recommended assessment for Ki67 because not all lesions contained 500 invasive carcinoma cells and evaluation of the hotspot was difficult in TMAs. Similarly to EZH2, we used the proportion score of Allred score to evaluate immunostaining of Ki-67. We show the immunostaining score of primary Ki-67 expression on the histogram. The histogram show the number of patients on the vertical axis, and immunostaining score in the horizontal axis. The median Ki-67 score across all PBC tumors sampled was 3. Ki-67 expression scores of 3, 4 and 5 were considered high expression. Ki-67 expression scores of 1 and 2 were considered low expression. In the St. Gallen Consensus meeting 2013, it was agreed that a threshold of ≥20% was clearly indicative of high Ki-67 expression [5]. Many studies reported that a cutoff for Ki-67 was 10-20% [6]. We considered that the evaluation of immunostaining of Ki-67 in this study was appropriate, because our cutoff for Ki-67 was >10%.

**
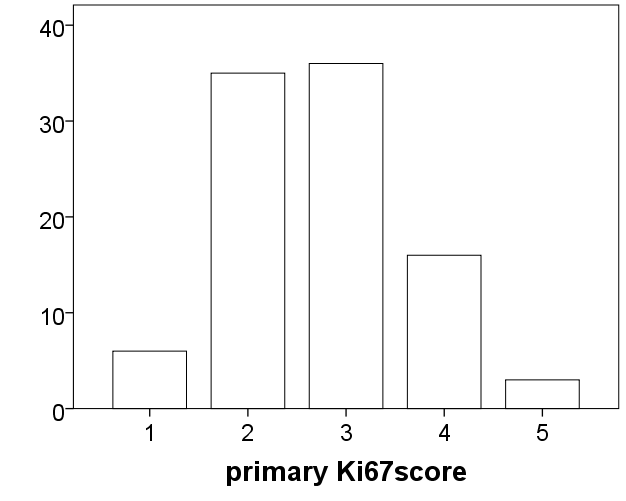
**

Patient number

1. Kleer CG, Cao Q, Varambally S, Shen R, Ota I, Tomlins SA, et al. EZH2 is a marker of aggressive breast cancer and promotes neoplastic transformation of breast epithelial cells. Proc Natl Acad Sci U S A. 2003; 100:11606–11611
2. Holm K, Grabau D, Lövgren K, Aradottir S, Gruvberger-Saal S, Howlin J,et al. Global H3K27 trimethylation and EZH2 abundance in breast tumor subtypes. Mol Oncol. 2012 Oct;6(5):494-506. doi: 10.1016/j.molonc.2012.06.002. Epub 2012 Jun 20.
3. Allred DC , Harvey JM, Berardo M, Clark GM. Prognostic and predictive factors in breast cancer by immunohistochemical analysis. Mod Pathol. 1998 Feb;11(2):155-68.
4. Dowsett M, Nielsen TO, A’Hern R, Bartlett J, Coombes RC, Cuzick J, et al. Assessment of Ki67 in breast cancer: recommendations from the International Ki67 in Breast Cancer Working Group. JNCI J Natl Cancer Inst. 2011;103(22):1656–64.
5. Goldhirsch A, Winer EP, Coates AS, Gelber RD, Piccart-Gebhart M, Thürlimann B,et al. Personalizing the treatment of women with early breast cancer: highlights of the St Gallen International Expert Consensus on the Primary Therapy of Early Breast Cancer 2013. Ann Oncol. 2013 Sep;24(9):2206-23. doi: 10.1093/annonc/mdt303. Epub 2013 Aug 4.
6. Horii R. The Japanese Breast Cancer Society Clinical Practice Guidelines for Pathological Diagnosis of Breast Cancer, 2015 Edition. Japan: Kanahara; 2015. p.243.
