# Supplementary material for: Expression of enhancer of zeste homolog 2 correlates with survival outcome in patients with metastatic breast cancer: exploratory study using primary and paired metastatic lesions
Source: BMC Cancer. 2017 Feb 27;17:160. doi: 10.1186/s12885-017-3154-3 (PMC5330119; doi:10.1186/s12885-017-3154-3)
Supplement: Additional file 3: Table S1. — Univariate and multivariate analysis of prognostic factors related to overall survival after recurrence including Ki-67 expression. (DOC 111 kb) [file 12885_2017_3154_MOESM3_ESM.doc]

**Additional file 3.**

**Table S1.** Univariate and multivariate analysis of prognostic factors related to overall survival after recurrence including Ki-67 expression (n = 96).

| Prognostic factor | Patients (*n*=96) | Univariate analysis | | | Multivariate analysis | | |
| --- | --- | --- | --- | --- | --- | --- | --- |
|  |  | HR | 95% CI | *P*-value | HR | 95% CI | *P*-value |
| Menopausal status, *n* (%) |  |  |  |  |  |  |  |
| pre- | 44 (45.8) | 1 |  |  |  |  |  |
| post- | 52 (54.2) | 1.396 | 0.891–2.186 | 0.145 |  |  |  |
| Tumor size, *n* (%) |  |  |  |  |  |  |  |
| ≤20 mm | 17 (17.7) | 1 |  |  |  |  |  |
| >20 mm | 77 (80.2) | 1.317 | 0.726–2.388 | 0.364 |  |  |  |
| unknown | 2 (2.1) |  |  |  |  |  |  |
| LN status, *n* (%) |  |  |  |  |  |  |  |
| positive | 63 (65.6) | 1.309 | 0.829–2.066 | 0.248 |  |  |  |
| negative | 33 (34.4) | 1 |  |  |  |  |  |
| Histological type, *n* (%) |  |  |  |  |  |  |  |
| ductal | 83 (86.5) | 1 |  |  |  |  |  |
| special^a^ | 13 (13.5) | 1.028 | 0.543-1.945 | 0.932 |  |  |  |
| LVI status, *n* (%) |  |  |  |  |  |  |  |
| positive | 63 (65.6) | 1.287 | 0.769–2.156 | 0.337 |  |  |  |
| negative | 25 (26.1) | 1 |  |  |  |  |  |
| unknown | 8 (8.3) |  |  |  |  |  |  |
| Operation status, *n* (%) |  |  |  |  |  |  |  |
| partial mastectomy | 17 (17.7) | 1 |  |  |  |  |  |
| mastectomy | 79 (82.3) | 0.894 | 0.500–1.596 | 0.704 |  |  |  |
| Chemotherapy, *n* (%) |  |  |  |  |  |  |  |
| adjuvant | 83 (86.5) | 1.320 | 0.707–2.461 | 0.383 |  |  |  |
| none | 13 (13.5) | 1 |  |  |  |  |  |
| Hormone therapy, *n* (%) |  |  |  |  |  |  |  |
| adjuvant | 50 (52.1) | 0.719 | 0.460–1.122 | 0.146 |  |  |  |
| none | 46 (47.9) | 1 |  |  |  |  |  |
| Primary ER status, *n* (%) |  |  |  |  |  |  |  |
| positive | 51 (53.1) | 0.541 | 0.344–0.850 | 0.008^*^ | 0.783 | 0.408-1.502 | 0.461 |
| negative | 45 (46.9) | 1 |  |  |  |  |  |
| Primary PR status, *n* (%) |  |  |  |  |  |  |  |
| positive | 47 (49.0) | 0.595 | 0.380-0.931 | 0.023^*^ | 0.938 | 0.491-1.791 | 0.846 |
| negative | 49 (51.0) | 1 |  |  |  |  |  |
| Primary HER2 status, *n* (%) |  |  |  |  |  |  |  |
| positive | 16 (16.7) | 1.122 | 0.616–2.046 | 0.706 |  |  |  |
| negative | 80 (83.3) | 1 |  |  |  |  |  |
| Primary Ki-67 expression, *n* (%) |  |  |  |  |  |  |  |
| high | 55 (57.3) | 1.848 | 1.170–2.917 | 0.008^*^ | 1.184 | 0.687-2.038 | 0.543 |
| low | 41 (42.7) | 1 |  |  |  |  |  |
| Primary EZH2 expression, *n* (%) |  |  |  |  |  |  |  |
| high | 54 (56.3) | 1.449 | 0.930–2.258 | 0.101 |  |  |  |
| low | 42 (43.7) | 1 |  |  |  |  |  |
| Metastatic ER status, *n* (%) |  |  |  |  |  |  |  |
| positive | 41 (42.7) | 0.972 | 0.625–1.513 | 0.901 |  |  |  |
| negative | 55 (57.3) | 1 |  |  |  |  |  |
| Metastatic PR status, *n* (%) |  |  |  |  |  |  |  |
| positive | 39 (40.6) | 0.692 | 0.440-1.089 | 0.112 |  |  |  |
| negative | 57 (59.4) | 1 |  |  |  |  |  |
| Metastatic HER2 status, *n* (%) |  |  |  |  |  |  |  |
| positive | 14 (14.6) | 1.282 | 0.673-2.440 | 0.450 |  |  |  |
| negative | 82 (85.4) | 1 |  |  |  |  |  |
| Metastatic Ki-67 expression, *n* (%) |  |  |  |  |  |  |  |
| high | 72 (75.0) | 2.422 | 1.394–4.206 | 0.002^*^ | 1.811 | 0.777-4.220 | 0.169 |
| low | 24 (25.0) | 1 |  |  |  |  |  |
| Metastatic EZH2 expression, *n* (%) |  |  |  |  |  |  |  |
| high | 59 (77.6) | 2.116 | 1.143–3.916 | 0.017^*^ | 1.215 | 0.490-3.010 | 0.675 |
| low | 17 (22.4) | 1 |  |  |  |  |  |
| Metastatic sites, n (%) |  |  |  |  |  |  |  |
| bones | 24 (25.0) | 0.899 | 0.548–1.476 | 0.674 |  |  |  |
| others | 72 (75.0) | 1 |  |  |  |  |  |
| Disease free interval |  |  |  |  |  |  |  |
| ≤2 years | 28 (29.0) | 1.713 | 1.086-2.702 | 0.021^*^ | 1.543 | 0.928-2.565 | 0.0094 |
| >2 years | 68 (71.0) | 1 |  |  |  |  |  |
| ≤10 years | 83 (87.0) | 1 |  |  |  |  |  |
| >10 years | 13 (13.0) | 0.861 | 0.442-1.679 | 0.661 |  |  |  |

**Abbreviations:** *CI*, confidence interval; *ER*, Estrogen receptor; *EZH2*, enhancer of zeste homolog 2; *HER2*, human epidermal growth factor receptor 2; *HR*, hazard ratio; *LN*, lymph node; *LVI*, lymphovascular invasion; *PR*, progesterone receptor

^a^Special type is invasive breast carcinoma except invasive ductal carcinoma.

^*^Indicates values that are statistically significant (*P*< 0.05).
